# Diagnostic Reboot: A Proposal to Improve Diagnostic Reasoning

**DOI:** 10.7759/cureus.12698

**Published:** 2021-01-14

**Authors:** Saqib Walayat, Benjamin Chaucer, Minchul Kim, Benjamin R Pflederer

**Affiliations:** 1 Gastroenterology, University of Illinois College of Medicine at Peoria, Peoria, USA; 2 Medicine, University of Alabama at Birmingham, Birmingham, USA; 3 Internal Medicine, University of Illinois College of Medicine at Peoria, Peoria, USA

**Keywords:** reboot, diagnosis, missed diagnosis, physical exam

## Abstract

Background

Diagnostic errors contribute to the morbidity and mortality of patients. We created and utilized a novel diagnostic tool (Diagnostic Reboot) and assessed its practical efficacy in the inpatient setting for improving diagnostic outcomes.

Design

This was a prospective sequential controlled study that involved University Hospitalist Adult Teaching Service (UHATS) teams. Senior residents were instructed to use the Diagnostic Reboot (DxR) tool whenever a patient aged 19-99 years was identified who had an uncertain diagnosis 24 hours into their admission.

Results

Participating residents identified a total of 32 patients as meeting the criteria of uncertain diagnosis after at least 24 hours of hospitalization during the six months of the study period. Of these, seven were during the intervention (DxR) period. The leading diagnosis was excluded in 3/7 (43%) patients in the DxR period and 13/25 (52%) in the control period. A new leading diagnosis was made in 6/7 (86%) cases in the DxR period and in 13/25 (52%) people in the control period. A new diagnostic plan was made in 100% of the patients in the DxR group and in 80% of patients in the control group. A new consultation was requested in 4/7 (57%) patients in the DxR group and in 9/25 (36%) patients in the control group. The Residents spent an average of 20 minutes on the DxR tool.

Conclusions

This study demonstrated that the use of DxR may help to improve analytical thinking in residents. It may also play a role in improving outcomes in medically challenging cases, but the use of the tool during the study period was not sufficient to draw concrete conclusions. The primary barrier to the use of such a diagnostic aid was identified as time pressure on a busy hospitalist service.

## Introduction

Diagnostic errors and uncertainty account for a significant amount of morbidity as well as increased mortality. They are a leading cause of medical malpractice claims and contribute to 6-17% of hospital adverse events [1]. The US Department of Health and Human Services Agency for Health Care Research and Quality identified diagnostic errors as an area of special focus in 2007 [2]. Diagnostic errors are usually due to cognitive bias. Cognitive bias encompasses a variety of unconscious influences, shortcuts, and behaviors that can influence one’s decision making [3]. There are numerous types of cognitive biases, including premature closure, anchoring, and confirmation bias. Diagnostic decisions are usually made by physicians under one of two major categories per dual-process theory: type 1, fast intuitive reasoning, and type 2, slow analytical reasoning. While type 1 fast intuitive reasoning is important and allows for efficient and accurate diagnostic thinking most of the time, it can lead to errors in more complex situations. Methods that encourage slow analytical reasoning have been proposed to improve diagnostic accuracy in certain situations, however, data from the real world, especially in inpatient clinical settings, is lacking. Previously, Sullivan et al. have used the mnemonic ‘SLOW’ to assess whether slowing down and thinking about a case resulted in improved diagnostic accuracy. Their study failed to show any difference in diagnostic accuracy, however, it did show that the physicians who were involved in the SLOW group had a better qualitative experience [3].

The purpose of this pilot study was to assess whether the application of a diagnostic tool (Diagnostic Reboot [DxR]) will improve diagnostic outcomes in an inpatient setting. Our two main aims are listed below. 

Aim 1: To assess whether enhancing analytic reasoning by using DxR can enhance diagnostic accuracy in an inpatient setting.

Aim 2: To assess medical team satisfaction after using DxR and identify barriers related to the use of the tool.

## Materials and methods

Research question

To assess whether a diagnostic aid such as DxR could enhance diagnostic accuracy and improve resident satisfaction in real-life inpatient settings for patients with an unclear diagnosis 24 hours into admission.

Study subjects

All senior residents (postgraduate year 2 and higher) on the internal medicine service at OSF St. Francis Medical Center were eligible for participation. Residents were recruited through an oral request during the noon conference and gave their informed consent to be included in the study.

Study design

**Figure 1:**
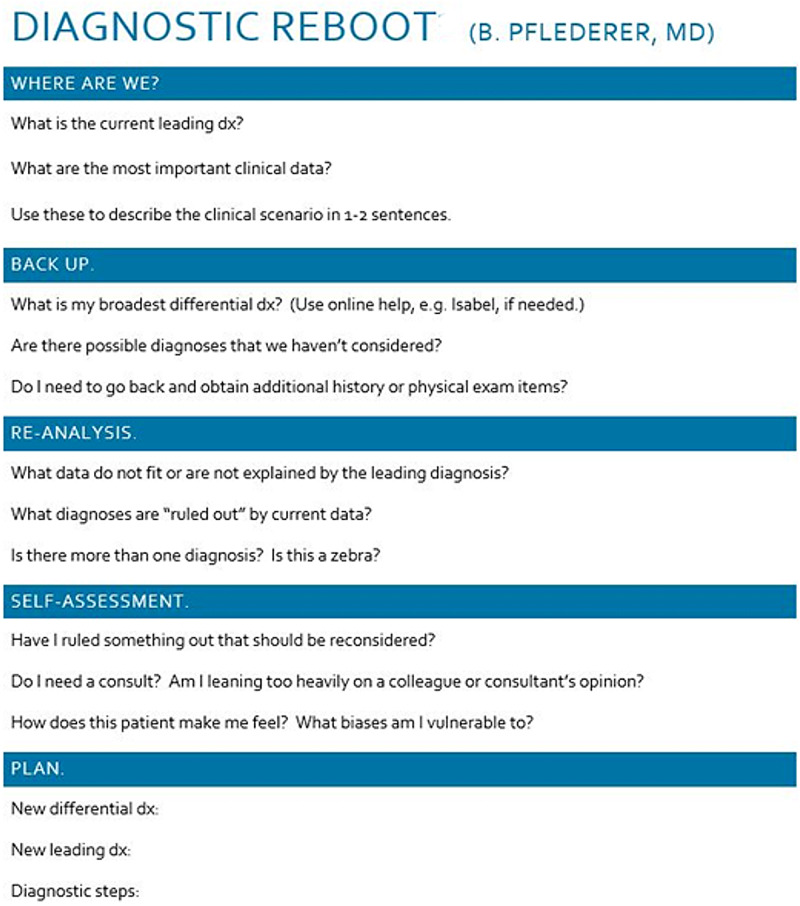
Diagnostic Reboot tool employed in our study Adapted from Trowbridge RL [9].

We conducted a prospective sequential controlled study of senior residents’ use of the DxR tool while caring for selected patients admitted to the University Hospitalist and Teaching Service (UHATS). The residents’ diagnostic reasoning for patients without a clear diagnosis after 24 hours of inpatient admission was the focus of the study. Attending physicians were rounding as per routine and were not aware whether the patient was involved in DxR. During the first three months of the study, patients without a clear diagnosis after 24 hours were identified and managed as per routine. After the initial three months (control period), the senior residents on teaching service were educated about the use of DxR, individually and collectively, via an afternoon conference. Subsequently, the senior residents identified patients without a clear diagnosis after 24 hours. During this period, the residents performed usual care but were also instructed to use the DxR to encourage more analytic reasoning. Log sheets were completed with information that included leading diagnosis on admission, time taken to perform DxR, and yes or no answer to the following questions: Was the previous leading diagnosis excluded? Was the new leading diagnosis made? Was the new diagnostic plan made? Was a new consultation requested? The Diagnostic Reboot tool used is shown in Figure 1.

The log sheets were secured in a locked container in the resident workroom. Each patient identified was assigned a number that was used on log sheets. The master data was locked and password protected. At the end of the study period (three months for the control period and three months for the intervention period), these log sheets were collected and the outcomes were assessed. A separate survey was given to all residents involved at the end of the study period to assess their satisfaction and difficulties with the use of diagnostic reboot. The study was approved by the local institutional review board.

Inclusion and exclusion criteria

Any senior resident (postgraduate year 2 and higher) who was on medicine floors was eligible for enrollment. The patient population of interest included adults (18-99 years) who were admitted to the adult medicine service and had an uncertain diagnosis after 24 hours in the hospital. Attending physician oversight and care of the patients was per routine, and attendings were blinded to the use of the DxR tool. Patient consent was not required, as the intervention was consistent with our standard education of residents in diagnostic reasoning, consistent with standard principles. Resident consent was obtained as mentioned above.

## Results

**Figure 2:**
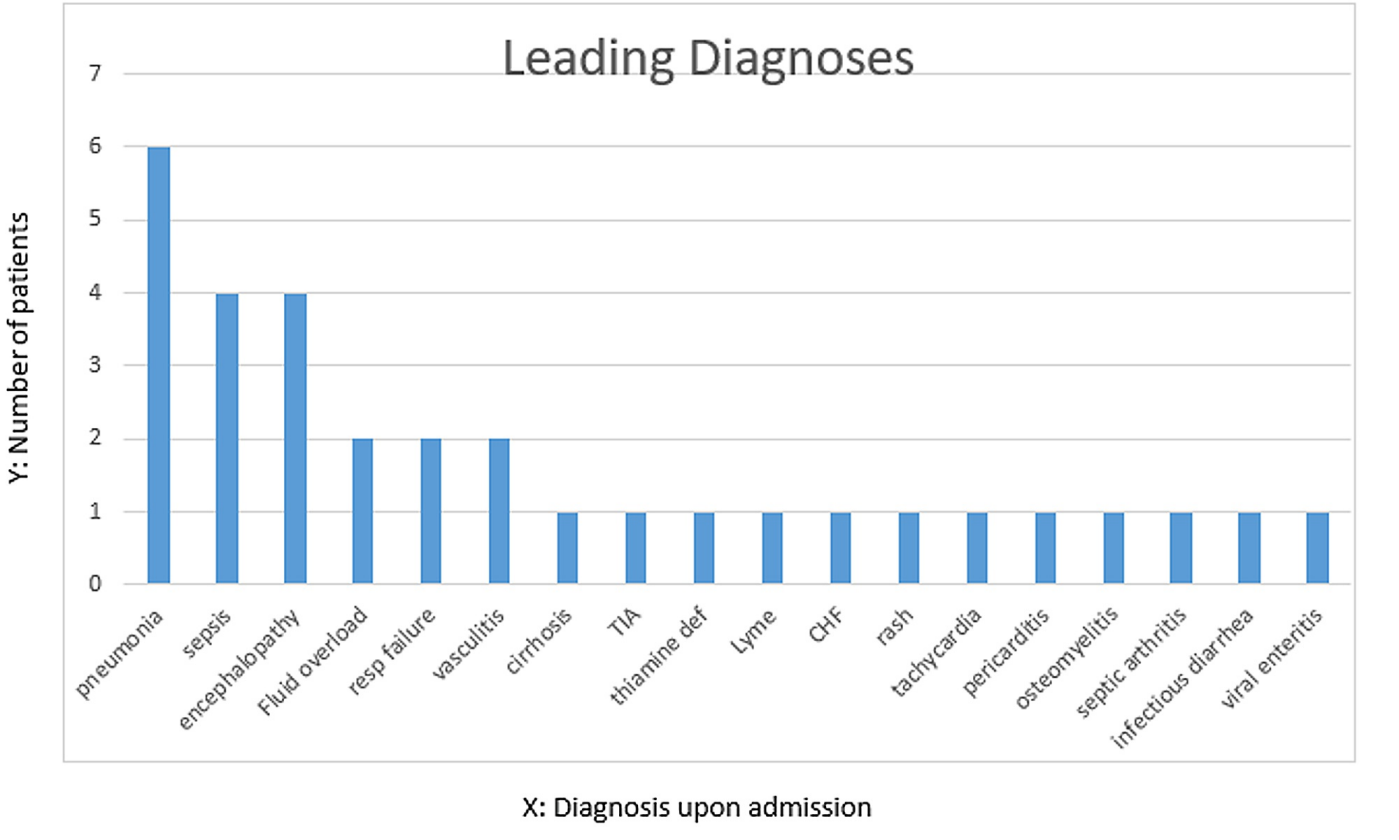
Leading diagnoses on admission The X-axis represents the leading diagnoses on admission while the Y-axis represents the number of patients with each diagnosis. CHF: congestive heart failure; TIA: transient ischemic attack

A total of 32 patients with uncertain diagnoses after 24 hours were identified during the six months of the study period. The mean age of these patients was 63 years. Twenty-five patients were selected during the initial control period and seven were selected during the intervention (DxR) period. The most common leading diagnosis for which Diagnostic Reboot was utilized included pneumonia, encephalopathy, and sepsis. Figure 2 shows the various leading diagnoses on admission. The residents required an average of 21 minutes to perform the Diagnostic Reboot, with time ranging from 10 minutes to 30 minutes.

The leading diagnosis was excluded in three patients (3/7; 43%) during the DxR period and in 13 patients during the control period (13/25; 52%). A new leading diagnosis was made in 6 out of 7 patients in the DxR period (6/7; 86%), (Table 1), and in 13 out of 25 patients in the control period (13/25; 52%). A new diagnostic plan was formulated in all patients who underwent diagnostic reboot, while a new diagnostic plan was eventually made in 80% of patients in the control period. A new consultation was requested in 4 out of 7 (4/7; 57%) patients in the Diagnostic Reboot period and in 9 out of 25 patients in the control period (9/25; 36%).

**Table 1:** Leading diagnosis on admission and upon discharge after diagnostic reboot in the intervention group

Patient No.	Leading Diagnosis on Admission	Leading diagnosis on discharge
1	Acute encephalopathy	Multifactorial encephalopathy
2	Community-acquired pneumonia	Hemorrhagic viral pneumonia
3	Pneumonia	Aspiration pneumonia
4	Ataxia	Multifactorial gait instability with cognitive decline
5	Sepsis	Infectious pneumonia
6	Acute respiratory failure secondary to pneumonia	Tracheal mass, pneumonia due to multidrug resistant organisms
7	Transient ischemic attack	Psychogenic syncope

In logistic regression analysis adjusting for age and gender, the proportion of change to new diagnosis in the control group was 53.10%, while that in the intervention group was 58.7%. Even though a trend was noticed towards the intervention group, the difference was not statistically significant (p = 0.817). The time to new diagnosis was 7.10 days in the control group and 5.87 days in the intervention group. This was not statistically significant with a p-value of 0.386.

**Figure 3:**
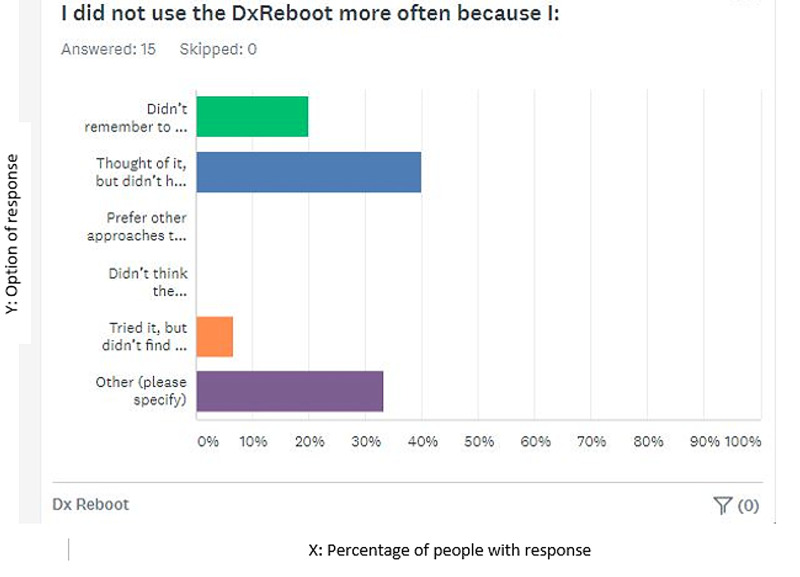
Various reasons mentioned by residents for not using Diagnostic reboot more frequently 1: Did not remember to do it, 2: Thought of it but did not have time, 3: Preferred other approaches, 4: Did not think the instructions were clear, 5: Tried but did not find it helpful, 6: Others). The X-axis represents the percentage of people with a particular response. The Y-axis represents the options of response.

A subsequent survey of the participating residents was carried out to assess each resident’s opinion and future research directions. Most of the residents reported having thought about the survey but did not have enough time to use the DxR or fill out the log sheet. Others did not use it because they did not think they had patients that met the criteria (uncertain diagnosis after 24 hours) during the study period, others reported doing it subconsciously, while some did not remember to do it (Figure 3).

## Discussion

Medical errors are reported to be the third leading cause of death in the United States [4]. These errors can occur at an individual level or at a system wide level [4]. The idea of a checklist to identify and rectify diagnostic errors has been around for some time but was popularized by Gawande in his book *The Checklist Manifesto: How To Get Things Right* in 2009 [5]. Previous studies have shown conflicting reports regarding the use of diagnostic aids. While some previous studies have shown residents to be influenced by decision aids [6], others have shown that physicians are less likely to use a decision aid to help with a diagnosis, even though it has been shown to improve diagnosis in patients [7]. While most of previous studies have used computer-based diagnostic aids or simulated patient care scenarios, our study was unique as we attempted to assess the implementation of a diagnostic aid tool in real-life clinical scenarios on medically challenging patients. Moreover, our diagnostic aid tool was different from previous ‘aids’ as its main purpose was to serve as a diagnostic reasoning tool (rather than just a simple checklist) to enhance and stimulate analytical thinking on medically challenging patients [8, 9].

Overall, even though limited by small numbers, our study show DxR may have a positive impact on clinical diagnostic reasoning, especially in medically challenging cases. Interestingly, in almost 90% of diagnostically challenging cases, a new leading diagnosis was made 24 hours into admission after using DxR, and a new plan was formulated in almost 80% of patients. This difference as compared to control could be due to enhanced cognitive thinking and mental stimulation aided by DxR, leading to new diagnostic formulations. Also, it could be because DxR serves as a checklist, allowing residents to spend an average of 20-30 minutes more on a challenging case in a step-wise and planned manner. This in turn could lead to a broader differential, 'connecting dots', and involvement of consultants on a more frequent and earlier basis to aid in diagnosis. Previously, Sibbald et al. have shown that checklists helped to find and fix mistakes in physicians of all levels, and physicians with minimal experience were shown to benefit more [10]. The mechanism for this is likely related to enhanced analytic scrutiny and a more careful examination of minor details [11]. Our findings support previous findings by Myung et al. that analytic reasoning reduced cognitive errors at least among novice physicians. Their study included medical students [12].

There were several limitations of our study. The most important is that the residents did not use the DxR for assessing enough patients to make a meaningful quantitative analysis. The reasons for this were various as mentioned in the resident survey, with the lack of time on a busy hospitalist service being the most important. Some residents did not remember to use it, and some mentioned doing it subconsciously but did not note the pertinent information on log sheets. Still others included residents not having enough patients meeting specified criteria and the short study duration. Another limitation was that it was senior residents performing the Diagnostic Reboot and going through the checklist, while some studies have suggested that more novice learners may benefit more from such cognitive reasoning tools [12,13]. Our results were also hampered by the limited number of patients being enrolled in the intervention group. This was due to the limited time duration of the study and not enough patients meeting the inclusion criteria. Moreover, knowledge gaps could have been different among different residents leading to differences in complexity.

The strength of our study included the study being performed in an actual clinical setting rather than with clinical vignettes.

Our study could serve as a building block on the use of diagnostic reasoning aids to improve diagnosis and decrease errors in medically challenging cases. The benefit of Diagnostic Reboot as compared to other tools is its simplicity and easy availability. It’s a simple card that can be stored in one’s pocket and can be used at any place, any time, even in resource-limited settings.

Future research directions should include steps to figure out a better way to encourage and facilitate the use of such tools by residents to enhance their analytical thinking. It would also be interesting to assess whether Diagnostic Reboot would be more helpful if used by interns, to assess whether Diagnostic Reboot leads only to a new diagnostic plan or if it alters the final diagnosis, and to assess whether residents who used diagnostic reboot have long-term changes in their thinking pattern. Future trials could be structured to assess whether such tools would increase or decrease the number of consultations, health care costs, and length of hospital stay in medically complicated patients.

## Conclusions

In conclusion, the diagnostic reboot can serve as a valuable tool applicable not only to senior residents but also to incoming interns and hospitalists to enhance cognitive thinking and potentially improve diagnostic reasoning on medically complicated patients. However, our study results were not significant to draw concrete conclusions due to limitations of sample size. Further studies are needed to determine the validity and usefulness of such tools in real-life clinical scenarios.
